# Hsa-miR-665 Is a Promising Biomarker in Cancer Prognosis

**DOI:** 10.3390/cancers15204915

**Published:** 2023-10-10

**Authors:** Xuefeng Guan, Krishna Chaitanya Pavani, Jayendra Chunduru, Bart J. G. Broeckx, Ann Van Soom, Luc Peelman

**Affiliations:** 1 Department of Veterinary and Biosciences, Faculty of Veterinary Medicine, Ghent University, Heidestraat 19, 9820 Merelbeke, Belgium; guan.xuefeng@ugent.be (X.G.); bart.broeckx@ugent.be (B.J.G.B.); 2 Department of Internal Medicine, Reproduction and Population Health, Faculty of Veterinary Medicine, Ghent University, Salisburylaan 133, 9820 Merelbeke, Belgium; krishnachaitanya.pavani@ugent.be (K.C.P.); ann.vansoom@ugent.be (A.V.S.); 3 Department for Reproductive Medicine, Ghent University Hospital, Corneel Heymanslaan 10, 9000 Gent, Belgium; 4 Department of Chemistry and Biochemistry, Texas Tech University, Lubbock, TX 79409, USA; jayendra.chunduru@ttu.edu

**Keywords:** Hsa-miR-665, cancer, biomarker, tumorigenesis

## Abstract

**Simple Summary:**

MicroRNAs (miRNAs) are small biomolecules that can indicate the presence of certain diseases, including cancer. They are stable and can be easily detected in blood and urine samples. Here we review the different roles of a specific miRNA, miR-665, in several cancer types and evaluate its potential as biomarker for early detection and prognosis concerning these cancer types. Considerable changes in miR-665 levels have been detected in several cancer types making miR-665 a good candidate biomarker for early detection of these cancers. However, miR-665 can be upregulated in one type of cancer and downregulated in another, and its levels can even change during the progression of the same cancer. Therefore, extrapolation between different cancer types is not advised and more clinical data are needed before miR-665 can be used as a biomarker for these cancers. A better knowledge of the functions of miR-665 will be valuable in improving early detection and treatment of cancer.

**Abstract:**

Biomarkers are biomolecules used to identify or predict the presence of a specific disease or condition. They play an important role in early diagnosis and may be crucial for treatment. MicroRNAs (miRNAs), a group of small non-coding RNAs, are more and more regarded as promising biomarkers for several reasons. Dysregulation of miRNAs has been linked with development of several diseases, including many different types of cancer, and abnormal levels can be present in early stages of tumor development. Because miRNAs are stable molecules secreted and freely circulating in blood and urine, they can be sampled with little or no invasion. Here, we present an overview of the current literature, focusing on the types of cancers for which dysregulation of miR-665 has been associated with disease progression, recurrence, and/or prognosis. It needs to be emphasized that the role of miR-665 sometimes seems ambiguous, in the sense that it can be upregulated in one cancer type and downregulated in another and can even change during the progression of the same cancer. Caution is thus needed before using miR-665 as a biomarker, and extrapolation between different cancer types is not advisable. Moreover, more detailed understanding of the different roles of miR-665 will help in determining its potential as a diagnostic and prognostic biomarker.

## 1. Introduction

A biomarker is a measurable indicator or characteristic that can be used to assess or evaluate biological processes, disease states, or responses to treatment. Biomarkers can be found in various biological samples such as blood, urine, tissue, or even genetic material like circulating tumor DNA (ctDNA). They can include molecules like proteins, enzymes, hormones, genetic material (DNA/RNA), or specific cellular characteristics. An ideal biomarker should ensure high-accuracy results and should be as minimally invasive as possible [1]. In addition, the earlier it can e detected in disease progression the more useful. For example, medical interventions such as surgical resection can enhance the survival chances more if tumors are detected before clinical symptoms appear or localized cancers have metastasized [2].

**Figure 1:**
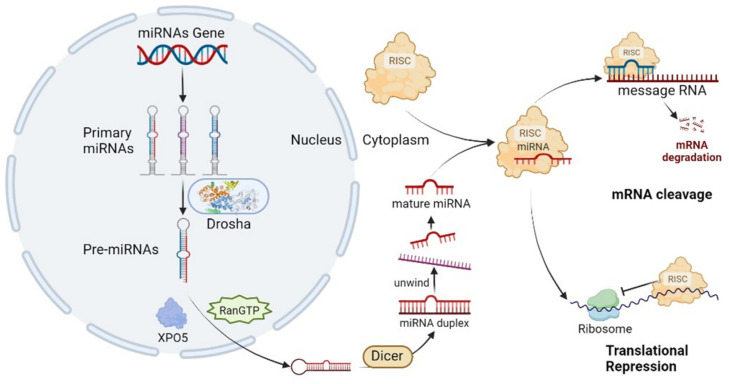
Overview of the biogenesis of microRNAs and the miR-RISC complex.

MicroRNAs are small endogenous RNAs derived from longer distinctive hairpin-forming precursors [3]. The precursors are first processed to Pri-miRNA, then trimmed by Drosha/DGCR8 (microprocessor) to Pre-miRNA, and these are transported to the cytoplasm and there cleaved by Dicer to double-stranded miRNAs. Only one of the strands is bound by Argonaute family proteins to form the miRNA-induced silencing complex (miRISC), post-transcriptionally silence its target, and shuttle between different subcellular compartments to control the rate of transcription and translation [4]. The functional miRISC complex then consists of a miRNA molecule and several protein components, including lipoproteins (low-density lipoprotein (LDL), high-density lipoprotein (HDL)), ribonucleoprotein complexes, Argonaute 1/2/3 (AGO1/2/3), and nucleophosmin 1 (NPM1) [5]. Once the RISC complex is formed, it recognizes and binds to complementary sites on the target mRNAs. This binding leads to either degradation of the target mRNA or repression of its translation into protein, depending on the degree of complementarity between the miRNA and the target mRNA [6]. Degradation or repression is mostly determined by a 6 to 8 ribonucleotide part of the miRNA known as the “seed” with which it binds to microRNA response elements (MREs) in target RNAs that are either perfectly or partly complementary. The seed is mostly situated at positions 2–8 from the miRNA 5′UTR end. Though usually miRNAs bind in the 3′UTR sequence of target mRNA, sometimes the MRE is situated in the 5′UTR or coding sequence. Individual miRNAs can target hundreds of different mRNAs and regulate many pathways. It is estimated that at least 60% of all human genes contain MREs (Figure 1) [7].

Identifying and using multianalyte panels that combine different classes of biomarkers into a single predictive score is advisable, since single biomarkers can be prone to interpatient variation and tumor heterogeneity, amongst other issues. An ideal panel would contain several types of biomarkers to buffer against inherent weaknesses of the individual classes. For example, secretion rates of protein biomarkers can vary significantly, whereas some biomarkers are only released once by dead cells and their detection can be masked by background shedding from healthy tissues [8]. MiRNAs are good candidates for inclusion in such a multianalyte panel for several reasons. Abnormal expression/levels have been shown to be associated with several cancer types. MiRNAs are stable molecules that can be secreted and taken up by cells. They are actively secreted in blood, urine, saliva, and other body fluids, which makes them easily accessible for disease screening and monitoring. MicroRNAs can be extracted and accurately quantified from minute sample quantities. These samples can be tiny amounts of blood, saliva, urine, and others, gathered by different methods like venous/interfinger blood collection and surgical excision/biopsy. Eventually, samples can be taken from tissue culture [9]. MiRNAs are relatively stable in the tumor microenvironment (TME) and can be carried by extracellular vesicles (EVs), which then participate in crosstalk between tumor cells [10,11]. They can also provide information on the molecular changes that occur early in cancer development, unlike many other biomarkers, such as proteins, which are often downstream indicators of disease and therefore may not detect the disease in its earliest stages [12]. All these aspects make miRNAs good candidates as (non-invasive) biomarkers.

Among several possible potential miRNAs that are available in cancer research, the highly conserved miRNA-665 is particularly interesting because it has been discovered to be dysregulated in a number of cancers. It was also one of the first miRNAs demonstrated to be directly regulated by the oncogene KRAS and by tumor suppressors TP53 and APC [13]. MiR-665 has been shown to be overexpressed in various types of tumors, including lung, breast, and liver, and has been associated with increased drug resistance [14,15,16]. Drug resistance in cancer refers to the phenomenon whereby cancer cells become unresponsive to therapeutic agents, leading to treatment failure and disease progression [17]. This can arise either through intrinsic mechanisms, such as genetic mutations, or acquired mechanisms, such as changes in cellular signaling pathways [18]. MiR-665 has been demonstrated to regulate key cellular signaling pathways, including the activation of survival signaling pathways and the modulation of drug efflux transporters. Additionally, miR-665 has been shown to promote tumor repopulation and the survival of cancer stem cells, thus contributing to the cancer acquiring drug resistance [19,20,21]. Therefore, miR-665 inhibitors can potentially be used as a therapeutic agent.

Here, we give an overview of the different roles of miR-665 known so far, and in that light evaluate its potential as a biomarker.

## 2. Signaling Pathways Regulated by miR-665

**Figure 2:**
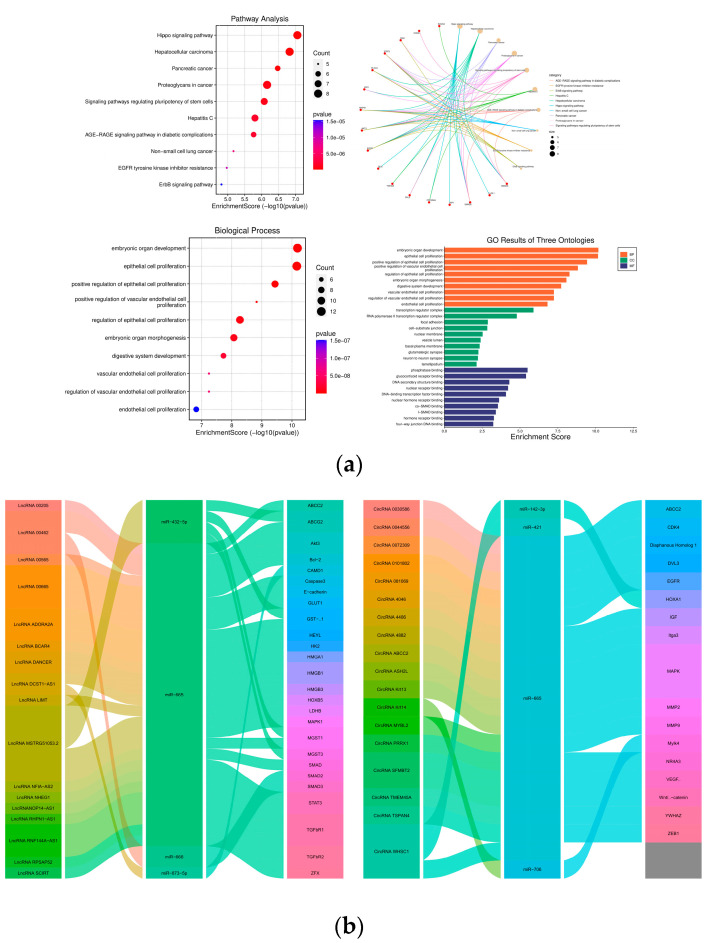
(**a**) Pathways and biological processes associated with Hsa-miR-665. Dot plot of the KEGG pathway enrichment analysis. The horizontal axis represents the gene ratio, while the vertical axis represents the enriched pathway name. The color scale indicates different thresholds of the *p*-value, and the size of the dot indicates the number of genes corresponding to each pathway. All RNAs were manually gathered from the publications we used in the manuscript. The data were analyzed using the R package clusterProfiler as provided online (https://www.bioconductor.org/packages/release/bioc/html/clusterProfiler.html (accessed on 5 May 2023)) (**b**) Pathway diagram of different ceRNA interactions with miR-665 as the node. All circRNAs/lncRNAs were manually gathered from the publications we used in the manuscript. The data were analyzed using the R package ggalluvial (https://github.com/corybrunson/ggalluvial (accessed on 6 June 2023)).

Understanding how miR-665 regulates various signaling pathways in cancer cells is crucial to determine its potential as biomarker for these cancers. MiR-665 has been implicated amongst others in tumor proliferation, migration, angiogenesis, apoptosis, and the activation of oncogenic pathways such as Mitogen-activated protein kinase (MAPK) and Wnt signaling [22]. Gene expression regulation by microRNAs is complex, due to the fact they can act on many targets; their effect is often subtle, as is their function in a network of other non-coding RNAs (ncRNAs). MicroRNAs can not only bind to mRNAs but also to long non-coding RNAs (lncRNAs), circular RNAs (circRNAs), and pseudogenes [23]. These molecules can contain several MREs and act as sponges for microRNAs and make them less available for target mRNAs, thus modulating their effect (Figure 2a,b).

### 2.1. MAPK Signaling Pathways

There are three canonical pathways within the MAPK network, indicated as ERK, JNK, and p38. They are activated by upstream signaling events, ultimately leading to the activation of GTPase or other protein kinases such as RAF [24].

As a central extracellular signaling sensor, ERK is a key player in chemotherapy-immune resistance in cancer because it activates pro-survival pathways in tumor cells that lead to cell proliferation, migration, apoptosis resistance, differentiation, and senescence, and modulates immune responses by affecting tumor-infiltrating immune cells and tumor-immune cell interaction [25]. MiR-665 regulates ERK signaling directly and indirectly and affects cell development and anti-apoptotic capacity. For example, in ovarian cancer, miR-665 targets SRCIN1, which inhibits SRC kinases to enhance the activation of MAPK/ERK and the mTOR axis to promote epithelial–mesenchymal transition (EMT) and cancer progression [26]. In gastric cancer cells, miR-665 is able to directly target and inhibit Cysteine Rich Transmembrane BMP Regulator 1(CRIM1) and MAPK1(ERK2) to decrease the proliferation of cancer cells [27,28]. The inhibition of miR-665 significantly increases the expression levels of glycolysis-related proteins GLUT1, LDH, and HK2, which in turn promote the proliferation and energy supply of cancer cells [29,30,31]. MiR-665 has also been implicated in the regulation of cell apoptosis in leukemia and bladder cancer [32] by hampering the activation of ERK1/2 [33]. Kaplan–Meier analysis showed that the median survival time of bladder cancer patients with higher miR-665 improved from 27.83 months to 57.27 months. In lung cancer, miR-665 can promote invasion and migration by targeting HEYL, a gene that encodes a transcription factor that inhibits Notch signaling, which suppresses p38 MAPK activity via induction of MKP-1. By suppressing HEYL expression, miR-665 can activate Notch signaling and induce EMT [34].

In addition to the three canonical MAPK pathways, there are also non-canonical MAPK pathways, such as MEK5/ERK5 and the TGFBR1/ERK/SMAD pathway [35]. These pathways are activated by different signals and involve different types of kinases. A study has shown that miR-665 can activate MEK5/ERK5 signaling and promote breast cancer metastasis by suppressing NR4A3, a nuclear receptor that can inhibit MEK5 expression and activity [19]. Although miR-665 acts in both cases on MAPK signaling, in breast cancer it acts as a promotor whereas in lung cancer it is a suppressor.

### 2.2. Wnt/β-Catenin Signaling Pathway

The Wnt signaling pathways are highly conserved signaling cascades that play critical roles in embryonic development, tissue homeostasis, and cell differentiation. In general, they are divided into canonical and non-canonical Wnt pathways. The canonical pathway, also known as the Wnt/β-catenin pathway, is activated by the binding of Wnt ligands to Frizzled receptors and LRP5/6 coreceptors, which leads to the stabilization and nuclear translocation of β-catenin. In the nucleus, β-catenin interacts with TCF/LEF transcription factors to activate target gene expression [36]. It is involved in cell fate determination, embryonic development, and tissue homeostasis. The non-canonical pathways do not involve β-catenin or LRP5/6 coreceptors. The non-canonical Wnt pathways can be further divided into two subtypes: the planar cell polarity (PCP) pathway and the calcium-dependent pathway. The PCP pathway regulates cell shape, polarity, and movement by activating small GTPases such as RhoA and Rac1. The calcium-dependent pathway modulates intracellular calcium levels and activates calcium-dependent enzymes such as protein kinase C (PKC) and calcineurin, which have been implicated in a wide range of biological processes, including stem cell self-renewal, tissue regeneration, and cancer development [37].

MiR-665 acts as a tumor-suppressive microRNA in neuroblastoma by directly targeting high mobility group box 1 (HMGB1) (activator of the Wnt/β-catenin pathway) and thus inactivating the Wnt/β-catenin pathway [38]. In lung squamous cell carcinoma (LUSC), miR-665 has been shown to target and inhibit TRIM8, which is a tripartite motif protein that inhibits the Wnt/β-catenin signaling pathway by degrading β-catenin. In this way, miR-665 promotes G1-S transition of the cell cycle and Wnt5a/β-Catenin signaling and activates the caspase-3 pathway, thus inhibiting LUSC apoptosis [20]. In testicular seminoma, miR-665 suppresses the expression of MKI67, a hub gene that promotes cell proliferation, migration, and invasion in testicular seminoma. This shows that miR-665 acts as a cancer inhibitor by downregulating the Wnt/β-catenin signaling pathways [39]. In large B-cell lymphoma (DLBCL), miR-665 has been shown to suppress the growth of cancer cells by targeting MYC and LASP1 [40]. MYC is a transcription factor that acts as a proto-oncogene in many cancers. It is activated by the Wnt canonical pathway and promotes cancer cell growth, especially in colon cancer. It has also been shown to be targeted by miR-665 in murine neuroblastoma cells, leading to inhibition of cell proliferation [41,42]. However, based on the available research, miR-665’s role in gastric adenocarcinoma is complex and context-dependent. While miR-665 often seems to act as a tumor suppressor, in gastric adenocarcinoma it has been shown to potentially promote cancer through the activation of the Wnt/β-catenin signaling pathway. This is believed to occur through miR-665’s ability to regulate SOCS3, leading to the activation of the FAK/Src signaling pathway, and potentially culminating in Wnt pathway activation [43,44]. The exact mechanism of how miR-665 promotes cancer in gastric adenocarcinoma remains unclear [45].

Overall, the interplay between the Wnt signaling pathways and miR-665 is complex, multi-faceted, and not always clear [46]. MiR-665 can regulate FAK (Focal adhesion kinase), Notch, MAPK, and Wnt pathways (and also some inflammatory response-related pathways which are not covered in this article). This crosstalk between two or more signaling pathways results in the involvement of countless proteins [22].

### 2.3. Regulation of miR-665

MicroRNAs can not only regulate many genes, but they are also themselves influenced by many different signals, including the level of miRNA transcription, the level of processing of Drosha and Dicer in the nucleus and cytoplasm, RNA editing, the level of RNA methylation, uridylation and adenylation, the level of Argonaute loading and the level of RNA degradation [47]. Importantly, their effect is influenced by the presence of other RNA types such as lncRNAs, circRNAs, and RNAs derived from pseudogenes, which can all contain MREs and compete for a miRNA according to the competing endogenous RNAs (ceRNAs) hypothesis [48]. These RNAs can serve as miRNA sponges, thereby repressing normal miRNA targeting activity on mRNA, making it important to deeply contextualize miRNAs before using them as therapeutic medication or relying on them as potential biomarkers and prognostic markers [49,50,51,52,53].

LncRNAs are non-protein coding transcripts longer than 200 nucleotides that play diverse roles in different physiological and pathological states [54]. They can control apoptosis, cell death, and cell proliferation, and regulate gene expression at various levels, including sponging of miRNAs [55]. Sponging of miR-665 by lncRNAs has been found in several different cancer types. For example, the lncRNA *MSTRG/SCIRT* can downregulate miR-665, thereby promoting lung cancer development [34,56,57]. Binding of lncRNA LINC00565 with miR-665 inhibits apoptosis and increases cell survival in gastric cancer [58]. LncRNA *NHEG1* has multiple MREs for miR-665 and can thus inhibit HMGB1, thereby suppressing neuroblastoma cell proliferation, migration, and invasion [38].

CircRNAs are single-stranded RNAs that form covalently closed loops. Their best-known function is as a miRNA-sponge [59]. For example, as previously described, ERK signaling is modulated by miR-665 through inhibition of NR4A3. However, this effect can be negated by the presence of *Circ_0030586*, which contains several MREs for miR-665 [60].

Pseudogenes are mostly non-coding segments of DNA that resemble functional genes. Most non-bacterial genomes contain many pseudogenes, often as many as functional genes [61]. They can regulate their parental transcripts by sponging shared microRNAs, thus acting as competing endogenous RNAs (ceRNAs). One example is PTENP1, a processed pseudogene that shares multiple predicted MREs [62] with PTEN, a well-known tumor suppressor which can be downregulated by miR-665. PTEN is a phosphatase that dephosphorylates the lipid PIP3 to PIP2, directly opposing the activation of the oncogenic PI3K/AKT/mTOR signaling network. The loss of function of the PTEN tumor suppressor is one of the most common events observed in many types of cancer [63].

More instances where ncRNAs regulate miR-665 levels will be given in the paragraphs describing the role of miR-665 in the specific cancer types.

## 3. The Roles of miR-665 in Human Cancers

Cancer is a malignant disease whose incidence increases with age. Its main cause can be attributed to the accumulation of damage to cells and their genetic content over time. According to pathological statistics, cancer has become the number one cause of death for people over 60 years old. With the advancement of medical technology, people’s life span has extended, making cancer become even more prevalent. According to the World Health Organization, more than 20% (around 2 billion) of the world’s people will be over 60 years old in 2050 [64].

**Figure 3:**
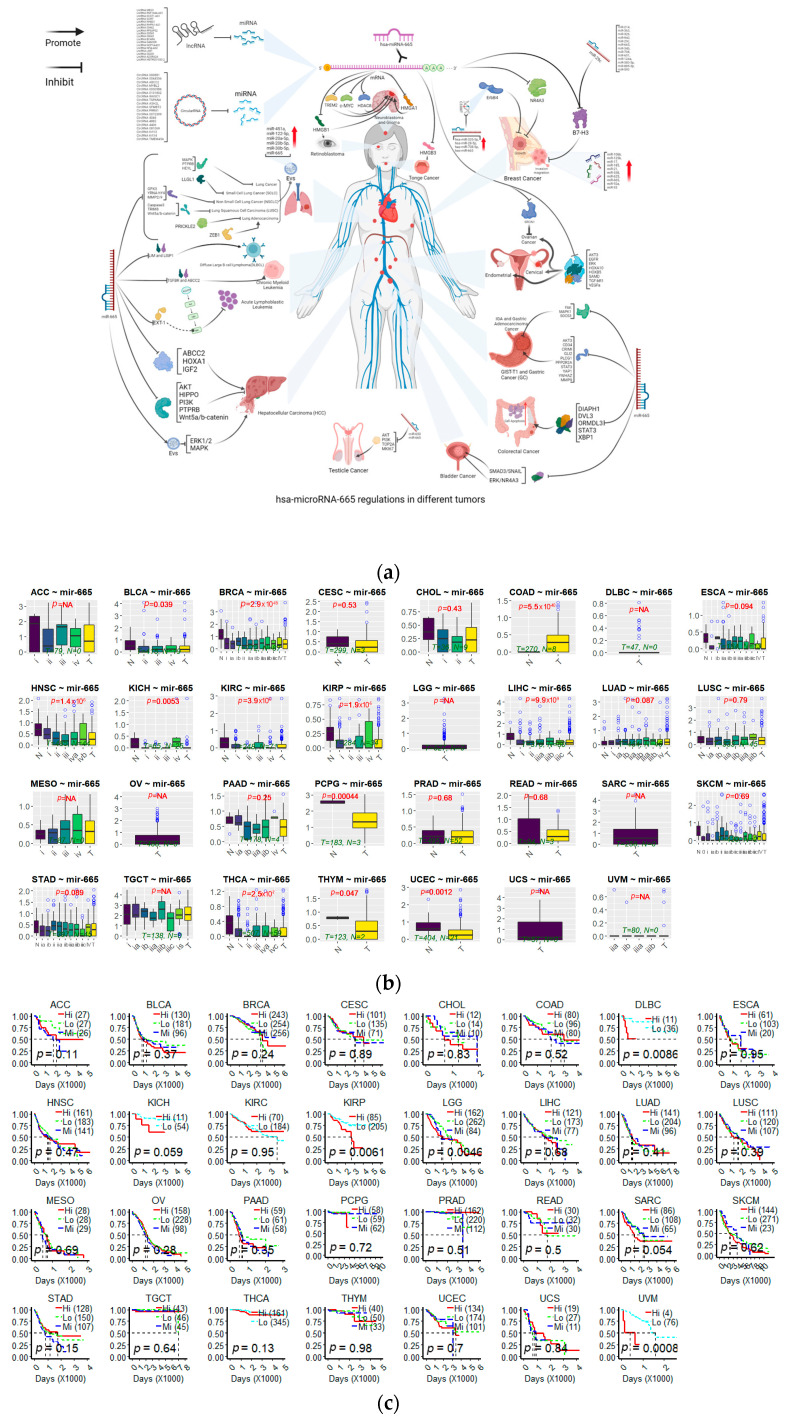
(**a**) Overview of different cancer types influenced by miR-665. The image was created using the online tool Bio Render. All data were collected manually from the articles used (https://www.biorender.com/ (accessed on 5 September 2023)). (**b**) MiRNA expression levels in normal and different tumor stages. (**c**) Survival curves of different miR-665 expression in the prognosis of different cancer patients. The miRActDB online tool provided by The University of Texas Health Science Center at Houston ([https://ccsm.uth.edu/miRactDB/ (accessed on 5 September 2023)) was used to create the image. The data used include 8375 patient samples across 31 cancer types and 20 normal tissues from the TCGA database, 941 cancer cell lines across 25 tissue origins in CCLE (https://depmap.org/portal/ (accessed on 5 September 2023)) and 11,688 samples across 30 healthy tissues in GTEx (https://www.gtexportal.org/home/datasets (accessed on 5 September 2023)).

Abnormal expression of miR-665 has been found in more than 20 cancer types. In most of the articles mentioned below, the involvement of miR-665 was found after addition or knockdown of some drug or a protein. In Table 1, an overview is given of the 65 target genes for which convincing data have been described that inhibition by miR-665 influences the development of at least one cancer type (Figure 3a). Of these target genes, inhibition of 28 genes promotes cancer development, while inhibition of the other 37 genes inhibits the growth of cancers. Some targeted genes are involved in different cancers and perform divergent functions in diverse cancers. Based on data from The Cancer Genome Atlas (TCGA) database, microRNA-665 expression is inconsistent in various cancers (Figure 3b) and the expression shows different survival curves of patients in different cancers (Figure 3c). The online-tool Kaplan–Meier Plotter (https://kmplot.com/analysis/ (accessed on 8 September 2023)) and data from the TCGA database were used to analyze survival curves [65,66]. In the following paragraphs, an alphabetically ordered overview is given of the types of cancers in which miR-665 dysregulation has been identified.

### 3.1. Breast Cancers

Breast cancer (BC) is one of the most common cancers affecting women globally, and it is the second most common cancer worldwide. Breast cancer has a significant impact on women’s health. To better understand BC and improve its prognosis, researchers have been studying different molecular and cellular mechanisms involved in the progression of BC. Some studies have shown that miR-665 overexpression in certain BC types, such as HER2-enriched and triple-negative breast cancer (TNBC), is associated with distant metastasis and poor prognosis [101,102].

MiR-665 promotes cancer growth, invasion, and metastasis by targeting NR4A3, thus activating the RAF/MEK/ERK pathway [19]. In light of this, miR-665 can be an interesting therapeutic target for BC by inhibition of NR4A3. Other studies have shown that miR-665 can also reduce the risk of death from BC by directly targeting B7-H3, an immunoregulatory protein that is overexpressed in several different cancer forms and that is often associated with metastasis and poor prognosis [103]. MiR-665 is one of the 13 known miRNAs that target B7-H3 directly by binding to its 3′-UTR region [70]. The expression of Epidermal Growth Factor Receptor 4 (ErbB4 or HER4) in BC is also regulated by miR-665. ErbB4 is a member of the ErbB subfamily of receptor tyrosine kinases which are important regulators of normal mammary gland physiology. ErbB4 expression can have both oncogenic and cancer-suppressive functions in BC [104,105,106]. High ErbB4 expression has been associated with a favorable outcome; however, the C allele of a T/C SNP (*rs1836724)* in the 3′ UTR of ErbB4 may lead to an unfavorable outcome. The rs1836724 SNP within the 3′-UTR of ErbB4 is in a binding site of four miRNAs (hsa-miR335-5p, hsa-miR-28-5p, has-miR-708-5p, and has-miR-665), and therefore can potentially influence binding of these miRNAs. The C allele was associated with more post-translational suppression corresponding to stronger mRNA binding by miR-665 and a more ER-positive phenotype in breast cancer. This means that higher levels of miR-665 will lead to a higher risk of breast cancer development in persons with the CC ErbB4 rs1836724 SNP genotype [107,108]. Kaplan–Meier analysis showed that BC patients with more miR-665 had a lower survival, with a reduction in upper quartile survival from 116.4 months to 76.53 months [65]. This suggests that miR-665 can be used as a negative prognostic biomarker for the survival time of breast cancer, eventually in combination with the *rs1836724* SNP in ErbB4.

### 3.2. Cervical Cancer

Cervical cancer is the fourth leading cause of cancer-related death in women [109]. It develops from the cells of the cervix, usually caused by infection with human papilloma virus (HPV). Regular pap smear screenings and HPV vaccination are important for early detection and prevention. Treatment options include surgery, radiation therapy, and chemotherapy. Symptoms may include abnormal vaginal bleeding, discharge, or pain during intercourse. With early detection and treatment, cervical cancer can be highly curable, but it remains a significant cause of cancer-related deaths in some parts of the world due to difficult access to medicines [110].

MiR-665 has been shown to inhibit cervical cancer malignant progress by targeting two cell surface receptors, EGFR and TGFBR1. EGFR is a member of the ErbB family of receptor tyrosine kinases that activate downstream signaling pathways regulating cell proliferation, survival, and differentiation. Overexpression of EGFR has been associated with cervical cancer development, invasion, and metastasis. TGFBR1 is a type I receptor for the TGF-beta family of growth factors, which activate downstream SMAD signaling pathways. TGFBR1 is also often overexpressed in cervical cancer, and has been implicated in the regulation of angiogenesis, invasion, and metastasis. EGFR expression is normally negatively regulated by miR-665 [111]. However, circ-*MYBL2*, a circular RNA spliced from the exons of the MYBL2 gene, highly expressed in cervical cancer cells, can act as a sponge for miR-665 and lift its inhibitory effect. Increasing the protein level of EGFR promotes the growth and drug resistance of cervical cancer cells [74]. As mentioned above, higher expression of EGFR/TGFBR1 has been shown to promote cervical cancer. As miR-665 is an inhibitor acting on this pathway it can be regarded as a tumor suppressor in cervical cancer development. Its (protecting) effect can however be taken away by the sponging capacity of lncRNA DANCR, which contains binding sites for miR-665 [35].

However, Kaplan–Meier analysis based on TCGA data showed that patients with higher levels of miR-665 had a lower survival, with the upper quartile survival going down from 36.1 months to 21.97 months. This contradiction may be due to the heterogeneity of the clinical TCGA data, in which different cancer types and research methods are mixed together, in combination with the complex regulatory mechanism of miR-665.

### 3.3. Circulatory Cancers

Circulatory cancers refer to a group of cancers that originate from the cells that form the circulatory system, including the blood and blood vessels. Some of the most common types of circulatory cancers are leukemia, lymphoma, and multiple myeloma [112,113,114,115].

Diffuse large B-cell lymphoma (DLBCL) is an aggressive form of cancer that affects B cells in any part of the body [116]. Studies have shown that miR-665 can suppress the progression of DLBCL by targeting and inhibiting LASP1 (LIM and SH3 protein 1) and MYC. LASP1, an actin-binding protein associated with actin assembly dynamics in cancer cells, which promotes invasion and metastasis, has been linked to metastatic breast cancer, hematopoietic tumors such as B-cell lymphomas, and colorectal cancer [40].

MiR-665 has been shown to be useful in the treatment of acute lymphoblastic leukemia (ALL) and chronic myeloid leukemia (CML) [117]. In ALL patients, miR-665 shows low expression and its addition promotes cancer cell apoptosis through targeting exotoxin glycosyltransferase 1 (EXT1), which functions in the Golgi apparatus and is involved in the synthesis of heparan sulfate, and the ERK1/2 signaling pathways [33]. TGFBR1 and ABCC2 (multidrug resistance-associated protein 2) are also targets of miR-665. ABCC2 plays an important role in transporting endogenous and exogenous substances. It reduces drug absorption, distribution, and excretion, reduces apoptosis and cycle arrest. and increases proliferation and drug resistance in CML cells [118]. Meanwhile, lncRNA *ADORA2A-AS1* is highly expressed in CML cells and can sponge miR-665 and negate its therapeutic effect [95].

The available evidence all seems to suggest that miR-665 can inhibit leukemogenesis and suppress tumor cell proliferation and invasion. So, miR-665 seems to be an interesting therapeutic target in circulatory cancers. The OncoLnc website analysis showed that patients with higher miR-665 levels exhibited higher survival rates, but the data were not statistically significant because the sample size was too low [119].

### 3.4. Endometrial Cancer

Endometrial cancer, also known as uterine cancer, is a cancer that arises from the lining of the uterus, typically caused by an imbalance between estrogen and progesterone signaling. Risk factors include obesity, hypertension, and estrogen exposure. Treatment involves surgical removal of the uterus and adjacent tissues, with or without adjuvant radiation or chemotherapy, and the prognosis is generally good if detected early and it has not spread beyond the uterus [120]. It can be treated with chemotherapy, using drugs such as carboplatin and paclitaxel [121]. MiR-665 targets HOXB5 to repress cancer progression [84]. HOXB5 is a member of the HOX family of transcription factors involved in regulating cell growth and differentiation. HOXB5 has been shown to be overexpressed in endometrial cancer, and its expression is correlated with tumor stage and poor prognosis. One way that HOXB5 may contribute to endometrial cancer is by promoting cell proliferation and survival. HOXB5 has been shown to upregulate the expression of several genes involved in cell cycle progression, such as cyclin D1, and to inhibit the expression of genes involved in apoptosis, such as caspase 3. HOXB5 may also interact with other signaling pathways that are involved in cancer development. For example, HOXB5 has been shown to activate the PI3K/Akt pathway, which promotes cell survival and growth, and to interact with the estrogen receptor, which is involved in regulating estrogen signaling in endometrial cancer [122]. In endometrial cancer, lncRNA *DCST1-AS1* attenuates the inhibitory effect of miR-665 on HOXB5 expression by taking up and reducing the amount of miR-665 and leads to increased HOXB5 levels [84].

The downregulation of HOXB5 by miR-665 is thought to be an important mechanism for regulating the growth and spread of endometrial cancer cells and may have implications for the development of new therapies for this disease. Kaplan–Meier analysis showed that patients with more miR-665 had a much lower survival, with a reduction in upper quartile survival from 108.37 months to 41.63 months. This suggests that miR-665 could serve as a significant biomarker for endometrial cancer.

### 3.5. Gastrointestinal Cancers

Gastrointestinal cancer refers to malignant conditions of the gastrointestinal tract (GI tract) and accessory organs of digestion, including the esophagus, stomach, liver, biliary system, pancreas, small intestine, large intestine, rectum, and anus. Obstruction, abnormal bleeding, or other associated symptoms can manifest themselves. Gastrointestinal cancers are responsible for more deaths than any other systemic cancer [123,124]. Early diagnosis and treatment are crucial for improving outcomes for patients with gastrointestinal cancer.

#### 3.5.1. Colorectal Cancer

Colorectal cancer (CRC) is a type of cancer that affects the colon and rectum, which are parts of the large intestine. It is the third most diagnosed cancer and the second leading cause of cancer-related death worldwide [64]. MiR-665 has been identified as a promising therapeutic target for CRC, due to its ability to regulate key oncogenic pathways. The expression of miR-665 is often downregulated in CRC, leading to an increase in the expression of its target gene Diaphanous Homolog 1 (DIAPH1) [72]. DIAPH1 has been shown to play a role in promoting tumor cell migration and invasion, making it a key contributor to CRC progression. In vitro studies have demonstrated that overexpression of miR-665 in CRC cells can effectively inhibit tumor cell proliferation, migration, and angiogenesis, while inducing apoptosis. This suggests that miR-665 has potent antiproliferative and proapoptotic effects in CRC cells. Additionally, the downregulation of miR-665 has been shown to increase the expression of Disheveled Segment Polarity Protein 3 (DVL3), a key regulator of the Wnt signaling pathway, which is often activated in CRC [73]. Overexpression of lncRNA BCAR4 promotes the development of colorectal carcinogenesis and maintains the properties of cancer stem cells by reducing the inhibitory effect of miR-665 on the transcriptional activator STAT3 through competitive inhibition. Cancer stem cells are cells with the capacity for self-renewal and multidirectional differentiation, capable of creating heterogeneity in tumors and leading to tumor recurrence and drug resistance. Therefore, lncRNA *BCAR4* may be a potential target for the diagnosis and treatment of colorectal cancer [93].

CircRNA-*004456/0101802* can also reduce the free level of miR-665, thereby increasing the expression of genes in the Wnt/β-catenin signaling pathway. The expression of these genes can increase cell cycle progression, inhibit apoptosis, and increase cell migration and invasion and angiogenesis, promoting the progression of colorectal cancer [67].

In conclusion, miR-665 has been shown to play a critical role in the regulation of key oncogenic pathways in CRC, including the Wnt signaling pathway and the regulation of stem cell self-renewal and differentiation [93]. Kaplan–Meier analysis showed that patients with higher miR-665 levels had a lower survival, with a reduction in upper quartile survival from 58.03 months to 36.53 months.

#### 3.5.2. Gastric Cancer

Gastric cancer (GC), also known as stomach cancer, is a type of cancer that affects the lining of the stomach [30]. It is a common and aggressive cancer with a high mortality rate. There are different subtypes of GC, including gastric adenocarcinomas, lymphomas, mesenchymal cancers, gastrointestinal stromal tumor (GIST), and signet-ring cell carcinoma. Despite various therapeutic strategies being developed for GC, the prognosis for patients with advanced GC remains poor, and treatments are often ineffective [64,125].

As mentioned in the paragraph about the MAPK signaling pathways, miR-665 has been shown to suppress the EMT and GC progression by targeting CRIM1 [28]. MiR-665 can also affect the activity of the MAPK signaling pathway by targeting the tyrosine protein kinase Met (c-MET). Downregulation of both genes inhibits the invasion and metastasis of tumor cells [124]. In addition, the downregulation of CRIM1 and c-MET can affect the expression of MMP2/9 (matrix metalloproteinase 2/9, an enzyme active in the degradation of the extracellular matrix), thereby reducing the impact of matrix degradation products in the tumor microenvironment [27,28,86]. However, miR-665 can activate the AKT/mTOR signaling pathway and promote tumor cell proliferation and anti-apoptosis activity by downregulating PPP2R2A (phosphatase 2A subunit B alpha isoform), a phosphatase subunit involved in the negative regulation of multiple signaling pathways. Low expression of PPP2R2A in gastric cancer tissues is associated with tumor staging, lymph node metastasis, and poor prognosis [88].

Yes-associated protein 1 (YAP1), a transcriptional co-activator and a core member of the Hippo signaling pathway, is an important target of miR-665 in GC. YAP1 is highly expressed in gastric cancer tissues, and promotes the growth, invasion, and drug resistance of tumor cells. Therefore, downregulation of YAP1 may induce apoptosis and autophagy in gastric cancer cells [98]. The effect of miR-665 can be counteracted by the presence of CircRNA-*100876* [58] or *PRRX1/SFMBT2* [126], another circRNA with miR-665 MREs that is often highly expressed in gastric cancer cells. MiR-665 can also inhibit the translation of TIMP3 by targeting its 3′UTR. TIMP3 is a metalloproteinase inhibitor, which can inhibit the hydrolysis activity of MMP by binding to it. Therefore, capturing of miR-665 by circRNA-*PRRX1/SFMBT2* results in the upregulation of TIMP3, thereby inhibiting the activity of MMPs and inhibiting the proliferation and invasion of cancer cells [127].

Downregulation of STAT3 by miR-665 inhibits the expression of pro-inflammatory factors, such as VEGF and IL-6, and enhances the immune system’s killing of tumor cells. This downregulation can be blocked by binding of lncRNA *RPSAP52* to miR-665. Since STAT3 can regulate the expression of multiple genes that promote proliferation, anti-apoptosis, angiogenesis, and immune escape in gastric cancer cells, presence of lncRNA *RPSAP52* enhances the malignant features of gastric cancer [94].

MiR-665 can also be captured by lncRNA *LINC00565*. This leads to upregulation of AKT3, one of three serine/threonine protein kinases of the AKT family, and to inhibition of apoptosis and increasing GC cell survival [58]. It was also found that LINC00565 is highly expressed in GC cells resistant to apatinib (an anticancer drug), while miR-665 was lowly expressed in these cells. A similar effect of LINC00565 was found in relation to the expression of MAPK1. MAPK1 is a signaling molecule that activates several tumor-associated pathways. Thus, LINC00565 promotes the proliferation, migration, invasion, and drug resistance of gastric cancer cells [27].

Clinically, miR-665 has been shown to be downregulated in gastric signet-ring cell carcinoma (GSRCC), in which the tumor cells contain a lot of mucus and show a ring-like structure, but in gastric adenocarcinoma, a malignant tumor originating from glandular cells within the gastric mucosa, miR-665 is upregulated. This suggests that miR-665 can inhibit GSRCC but promote gastric adenocarcinoma [76].

MiR-665 inhibits metastasis and chemoresistance in GSRCC by downregulating its target genes, GLI2 and PLCG1. Zinc-finger transcription factor GLI2 activates the Hedgehog signaling pathway to promote GSRCC tumor invasion and induce chemoresistance. The PLCG1 isoform of phospholipase (PLC) promotes tumor metastasis by triggering a number of signaling pathways. including the PKC signaling pathway, the calcium signaling pathway, Ras/ERK, the VEGF signaling pathway, and the MAPK pathway; it also promotes upregulation of the MDR1 gene and induces tumor cell resistance to chemotherapeutic agents [76]. CircRNA *Paired-Related Homeobox1* promotes GSRCC progression via downregulating microRNA-665 which inhibits YWHAZ expression [100].

MiR-665 promotes the progression and invasion of gastric adenocarcinoma by targeting SOCS3. SOCS3 is a cancer suppressor that is lowly expressed in some types or subtypes of gastric cancer tissues. It regulates tumor proliferation, invasion, and metastasis. Downregulation of SOCS3 may attenuate the inhibition of signaling pathways such as JAK/STAT and EGFR, promoting tumor cell growth and drug resistance. LncRNA MEG3 can potentially slow down the progression of gastric adenocarcinoma by sponging miR-665 [45].

In gastrointestinal stromal cancers (GISTs), the expression of CD34 (associated with tumor angiogenesis) varies depending on the location within the gastrointestinal tract where the GIST arises. For example, higher levels of CD34 expression have been observed in the GIST-T1 subtype, which arises in the stomach, compared to GISTs that arise in the small intestine. This expression pattern seems to be inversely correlated with the expression of miR-665 [71]. These authors also demonstrated experimentally that CD34 can be targeted and downregulated in GISTs by miR-665 and so decrease vascular density in the tumor microenvironment. This impairs tumor nutrient supply and metastatic capacity and proliferation of GISTs.

In conclusion, there have been many studies looking at the role of miR-665 in gastric cancer. MiR-665 is expressed at different levels in different types or subtypes of gastric cancer tissues. It can affect the proliferation, migration, invasion, apoptosis, autophagy, angiogenesis, and drug tolerance of GC tumor cells by targeting several downstream genes. Kaplan–Meier analysis showed that gastric adenocarcinoma patients with more miR-665 had a lower survival, with a reduction in upper quartile survival from 60.37 months to 25.97 months. This suggests that miR-665 can potentially serve as a biomarker for gastric adenocarcinoma, while no significant differences in survival curve were observed for the other GC subtypes.

#### 3.5.3. Hepatocellular Carcinoma

Hepatocellular carcinoma (HCC), also known as liver cancer, is a leading cause of cancer-related death. MiR-665 has been shown to be highly expressed in HCC. This leads to the downregulation of genes like *PTPRB* [128], a cancer suppressor, and *HOXA1* [82], which suppresses HCC cell proliferation and metastasis [85]. MiR-665 also regulates the level of the EMT-related protein LIMT in HCC, which can increase sorafenib resistance (a common treatment for HCC) [129]. This, in combination with the fact that the expression of miR-665 in HCC can be easily detected in venous blood serum, makes it an interesting potential non-invasive biomarker for the disease. This is corroborated by the fact that miR-665 shows an elevated expression in exosomes of HCC cancer cell lines like HepG2, PLC, and Hep3B [75]. The increased expression of miR-665 in exosomes has been linked to the malignant potential of HCC, as it promotes cancer cell proliferation. It should be noted that the progression of the disease is also influenced by other factors, such as chronic viral hepatitis, exposure to toxins, metabolic disorders like type 2 diabetes, and congenital disorders. Because the possibility exists that miR-665 is also involved in these diseases, it might be difficult to pinpoint the source of variation in miR-665 expression [130]. Another complicating factor might be the expression of the lncRNA *LIMT*, which can act as a sponge for miR-665, preventing it from binding to two of its important mRNA targets, MAP4K3 and CDH13. MAP4K3 (mitogen activated protein kinase kinase-kinase-kinase 3) is a kinase involved in a variety of signaling pathways, including mTORC1 (mammalian target of rapamycin complex 1) and AMPK (AMP activated protein kinase). It has anti-cancer effects in HCC, inducing apoptosis and autophagy, and reducing drug resistance. CDH13 (cadherin 13) is a member of the family of calcium-dependent adhesion molecules involved in cell adhesion and migration. In hepatocellular carcinoma, miR-665 can promote cancer cell metastasis, enhance anti-apoptotic ability, and increase the drug resistance of hepatocellular carcinoma cells to sorafenib by inhibiting CDH13. Therefore, lncRNA LIMT can be seen as a tumor suppressor by preventing miR-665 from binding to the mRNAs of the two anti-cancer genes, MAP4K3 and CDH13 [129]. Finally, the circRNAs *ABCC2*, *TMEM45A*, and *WHSC1* can decrease the occurrence of hepatocellular carcinoma by binding to miR-665, so reducing the level of free miR-665 and reducing levels of cancer-promoting factors such as p53 and p21 [67,82,85].

Kaplan–Meier analysis showed that patients with higher miR-665 levels had a lower survival curve, with a reduction in upper quartile survival from 76 months to 51 months in the HCC database. This indicates that miR-665 can serve as a biomarker for hepatocellular carcinoma. However, it should be mentioned that in a pan-cancer study (372 patients) [65], the survival period of patients with high miR-665 levels was significantly increased (from 27.57 months to 81.87 months), which seems to indicate that miR-665 generally acts as a tumor suppressor in liver cancer.

### 3.6. Glioma Cancer

Glioma originates from glial cells that support and nourish neurons. There are different types, such as astrocytoma, oligodendroglioma, and ependymoma. These tumors can be benign or malignant. Symptoms include headaches, seizures, speech difficulties, and unilateral weakness or numbness. Treatment options, including surgery, radiation, and chemotherapy, depend on the tumor type and stage [131].

The regulatory functions of miR-665 in glioma cancer seem mainly focused on the HMG protein family. Overexpression of miR-665 in glioma cells inhibits tumor cell proliferation, migration, and invasion by targeting high mobility group box 1 (HMGB1) and High Mobility Group AT-hook 1 protein (HMGA1), deactivating the Wnt/β-catenin pathway [80]. As a key protein that promotes tumor angiogenesis and that regulates the immune response, HMGB1 seems to be the main target by which miR-665 influences metastasis and invasion. The effect of miR-665 reducing HMGA1 can be inhibited by lncRNA *144A-AS1* [79].

Kaplan–Meier survival analysis showed that patients with high expression of miR-665 had a longer overall survival time than the low-expression group [80]. This indicates that miR-665 acts as a tumor suppressor in glioma, is a good candidate prognostic biomarker, and may be used together with HMGB1 as a novel therapeutic target.

### 3.7. Lung Cancers

Lung cancer is a leading cause of cancer-associated mortality worldwide, with small-cell lung carcinoma (SCLC) and non-small-cell lung carcinoma (NSCLC) being the two major forms [132,133]. SCLC is a fast-paced and aggressive form of lung cancer, with a low survival rate of approximately 7% [134]. The expression of miR-665 was found to be significantly higher in SCLC tissues compared to normal non-cancer tissues. MiR-665 can promote the proliferation of SCLC cells by binding to the 3′ UTR of target protein gene *LLGL1* (LLGL Scribble Cell Polarity Complex Component 1) and inhibit its expression. LLGL1 is a protein component of the cytoskeletal network that associates with non-muscle myosin II heavy chain. Compared with non-tumor normal tissues, the expression of LLGL1 was significantly lower in SCLC tissues [135].

NSCLC is the most frequent type of lung cancer. Its invasion rate is not as fast as that of SCLC. The 5-year survival rates for NSCLC range from 73% in stage IA disease to 13% in stage IV disease, making it a significant global health burden [136]. Studies have shown that miR-665 is upregulated in NSCLC cancer tissues and plasma of patients and can target tyrosine phosphatase receptor type B (PTPRB) to enhance cell proliferation, migration, and invasion in vitro [90,99]. *MSTRG.51053.2*, a novel lncRNA, can regulate miR-432-5p and miR-665 to form an endogenous RNA competition network to regulate the drug resistance of NSCLC cells to cisplatin (cis-diamminedichloroplatinum II (CDDP)). MiR-665 can reduce the drug resistance of NSCLC cells to CDDP by targeting ABCG2 and cooperating with the drug to inhibit ABCG2 expression. It also indirectly regulates *MGST1*, *MGST3,* and *GST-ω1*, three other genes associated with CDDP resistance. *MGST1, MGST3,* and *GST-ω1* are glutathione S-transferases (GSTs), which are important enzymes involved in the detoxification process. They can catalyze the binding of substrates to glutathione, thereby reducing the toxicity of drug metabolites to cells. The expression levels of these genes and proteins were found to be significantly upregulated in CDDP-resistant NSCLC cells, while CDDP resistance was reduced after interfering with them with siRNA [56]. On the other hand, miR-665 can also be regulated by circRNAs. CircRNA-0078767 can increase the expression of GPX3 by binding with miR-665. GPX3 is an antioxidant enzyme, which can reduce oxidative stress, increase apoptosis, and reduce cell migration and invasion, thereby inhibiting the occurrence of NSCLC [77].

In patients with lung adenocarcinoma (LUAD), a subtype of NSCLC, miR-665 can enhance the malignancy of cells by binding to Prickle Planar Cell Polarity Protein 2 (PRICKLE2). PRICKLE2 can inhibit the migration and invasion of lung adenocarcinoma cells, as well as the formation of cancer stem cells. The effect of miR-665 on PRICKLE2 can be counteracted by circRNA-000881, which sponges miR-665 [89]. LncRNA *MSTRG/SCIRT* promotes exosome loading of miR-665 with the help of hnRNPA1 (heterogeneous nuclear ribonucleoprotein A1). It is a protein that plays a key role in the regulation of alternative splicing. Thus, lncRNA *MSTRG/SCIRT* contributes to lung cancer progression, potentially providing a novel diagnostic and therapeutic target [34,56].

The role of miR-665 in NSCLC is complex and still not fully understood. MiR-665 appears to enhance the invasiveness of cancer cells and promotes lung cancer. Kaplan–Meier analysis showed that, in NSCLC, patients with more miR-665 expression exhibited better survival in LUAD, with the median survival curve improving from 38.1 to 71.1. This makes miR-665 an interesting candidate biomarker for LUAD. However, in another subtype of NSCLC, LUSC (see also Section 2.2), that accounts for more than 30% of NSCLC patients [20], miR-665 did not have a significant effect on patient survival, with median survival levels hardly changing from 49.97 to 49.73 [20,65].

### 3.8. Neuroblastoma

Neuroblastoma is a tumor that arises from immature nerve cells called neuroblasts. It is most prevalent in young children, typically under the age of five, and often originates in the adrenal glands located above the kidneys. However, it can also occur in the nerve tissue in the neck, chest, abdomen, or pelvis. The etiology of neuroblastoma is not completely understood, but certain genetic mutations and chromosomal abnormalities have been identified as risk factors. Symptoms of neuroblastoma vary depending on the tumor’s size and location, but may include a mass or lump in the abdomen, chest, or neck, bone pain, fatigue, and fever. Treatment options for neuroblastoma are determined by the stage of the cancer and the child’s age, and may include surgery, chemotherapy, radiation therapy, immunotherapy, or a combination of these modalities. Early detection and treatment offer the best prognosis [137,138].

In neuroblastoma, miR-665 acts as a tumor suppressor by inhibiting HMGB1 and thus blocking the Wnt/β-catenin pathway [38]. The influence of miR-665 on HMGB1 can be counteracted by the presence of lncRNA *NHEG1*, sponging the microRNA. NHEG1 has several MREs for miR-665 and can compete with HMGB1, which affects the development and resistance of NB. For HMGB1, it induces autophagy in nerve sheath cells (Schwann cells) through the classical Beclin 1-mediated pathway and promotes co-culture proliferation of NB cells with nerve sheath cells [139]. HMGB1 also reduces oxidative stress and apoptosis in NB cells caused by chemotherapeutic agents such as doxorubicin [140]. Furthermore, miR-665 has been found to activate cell death and increase the number of cells in G1 phase and decrease the cell number in S phase by targeting HDAC8 and c-MYC, reducing the malignancy of mouse neuroblastoma. Histone Deacetylase 8 (HDAC8) promotes cell proliferation, migration, and invasion, and activates the PI3K/Akt and Wnt/β-catenin pathways. C-MYC promotes the expression of genes involved in cell cycle progression and cell survival, and activates the PI3K/Akt and MAPK/ERK pathways [41]. HDAC8 and c-MYC play important roles in promoting the growth and spread of neuroblastoma cells. LncRNA *NHEG1* could sponge miR-665 which inhibits HMGB1 that promotes neuroblastoma, so neuroblastoma tumor progression is upregulated by the lncRNA *NHEG1* [38,79].

Kaplan–Meier analysis showed no significant effect of miR-665 on patient survival curves in neuroblastoma [38].

### 3.9. Ovarian Cancer

Ovarian cancer is an often-fatal type of cancer. It is the fifth leading cause of cancer-related deaths in women [141]. Symptoms of ovarian cancer may include abdominal bloating or swelling, quickly feeling full when eating, weight loss, discomfort in the pelvic area, fatigue, back pain, changes in bowel habits such as constipation, and a frequent need to urinate [142].

In ovarian cancer, miR-665 targets several important genes involved in cell proliferation, migration, invasion, and angiogenesis. They include AKT1/2/3, a serine-threonine protein kinase that promotes cell survival and growth, Cyclin D1, a protein that controls the progression of the cell cycle, p21, a protein that inhibits cell cycle progression, MMP9, an enzyme that helps break down the extracellular matrix, allowing cancer cells to invade and metastasize, and HOXA10, a transcription factor that regulates the expression of many genes involved in cell growth and differentiation. By targeting these genes, miR-665 can suppress the proliferation, migration, and invasion of cancer cells, as well as reduce the formation of new blood and lymphatic vessels that are necessary for cancer growth and metastasis. Additionally, miR-665 can regulate the expression of VEGFA, another protein that promotes angiogenesis and lymph angiogenesis. By inhibiting VEGFA expression, miR-665 can further reduce the growth and spread of cancer cells [68,83]. *Circ-ASH2L* can compete with miR-665, thereby reducing the inhibition of VEGFA by miR-665, increasing the level of VEGFA, and promote the formation of blood vessels and lymphatic vessels and tumorigenesis [97].

LncRNA *DANCR* can promote ovarian cancer through the MAPK/ERK/SMAD pathway. An interesting point is that, in cardiovascular diseases, miR-665 also regulates the MAPK/ERK pathway by targeting TGFBR1 and promotes apoptosis of vascular epithelial cells. These observations suggest that miR-665 inhibits the proliferation of cancer cells via targeting TGFBR1 [143].

However, in contradiction to the above, Kaplan–Meier analysis based on TCGA data showed that patients with higher miR-665 levels had lower survival curves, with the lower quartile survival reduced from 55 to 42.63 months. The only experimental evidence supporting the TCGA data is described by Ping Zhou et al. [26]. Their data suggest that miR-665 targets SRCIN1 and the inhibition of miR-665 promotes SRCIN1 expression, leading to progression of ovarian cancer.

### 3.10. Retinoblastoma

Retinoblastoma is a cancer that affects the retina, the light-sensitive layer of cells in the back of the eye. The disease may affect one or both eyes, and can be caused by genetic mutations that affect the *RB1* gene, which normally suppresses tumor growth. Common symptoms of retinoblastoma include a white or yellowish glow in the pupil (leukocoria), crossed eyes, poor vision, or redness and swelling of the eye. Treatment for retinoblastoma depends on the stage and extent of the cancer, and may include chemotherapy, radiation therapy, cryotherapy, laser therapy, and/or surgery to remove the affected eye(s) if necessary. Early detection and treatment can preserve vision and prevent the spread of the cancer [144].

Additionally, HMGB1 may interact with other signaling pathways that are involved in retinoblastoma development and progression, such as the PI3K/AKT and MAPK/ERK pathways. By activating these pathways, HMGB1 can promote cell survival, proliferation, and migration, and may contribute to the aggressive behavior of retinoblastoma cells [81]. Addition of miR-665 inhibitor to retinoblastoma Y79 and WERI-RB-1 cell lines increased HMGB1 and LASP1 expression at both mRNA and protein levels [145]. This suggests that miR-665 is able to inhibit HMGB1 and thus cancer progression.

Due to insufficient numbers of clinical samples, there are currently no statistically significant survival curve data available.

## 4. The Effect of miR-665 on Drug Resistance

Cancer drug resistance is a major challenge in the treatment of cancer. It refers to the ability of cancer cells to evade the therapeutic effects of chemotherapy drugs, leading to treatment failure and disease progression. The development of resistance to chemotherapy drugs is a complex process that is influenced by various genetic and epigenetic changes in cancer cells. In addition to being a marker of patient prognosis after chemotherapy, it has been shown that miR-665 can enhance the specificity and effectiveness of drugs and reduce resistance to drugs such as paclitaxel through EGFR/HOXB5, cisplatin through ABCG2, imatinib through ADORA2A, sorafenib through CDH13/HOXA1, and apatinib through VEGF [95,129,146]. The level of miR-665 expression has been shown to be negatively correlated with the prognosis for patients with cervical cancer, and it can also be used as a predictor of resistance to neoadjuvant radio-chemotherapy. That means the role of miR-665 in cancer therapy is not limited to inhibiting the proliferation and invasion of tumor cells; it can also modulate tumor resistance to drugs and radiation by several methods.

Firstly, miR-665 can increase the sensitivity of tumor cells to chemotherapeutic agents by inhibiting the expression of certain drug target genes. For example, miR-665 can increase the sensitivity of non-small cell lung cancer cells to chemotherapeutic drugs by downregulating the expression of ABCG2. Upregulation of ABCG2 can also be induced by the sponging effect of lncRNA *MSTRG.51053.2* on miR-665 [56]. In gastric cancer, the sensitivity of antitumor drugs can be improved by miR-665 by influencing the expression of drug pumps (like P-glycoprotein) and metabolism-related proteins (like GLUT1, LDHB, and HK2). This effect of miR-665 is achieved by its inhibition of the MAPK1 signaling pathway. However, this effect can be countered by overexpression of LINC00665, which promotes cell proliferation and anti-apoptosis activity by upregulating the expression of MAPK1 [27].

Secondly, miR-665 can also increase the sensitivity of tumor cells to therapy by regulating cellular DNA repair mechanisms. For example, in LUSC, miR-665 can reduce tumor cell tolerance to drug therapy by downregulating the expression of the DNA repair gene TRIM8 [20].

Finally, miR-665 can also modulate tumor resistance and drug resistance by affecting the tumor microenvironment. For example, in gastric cancer, inhibition of the miR-665/MAPK1 signaling pathway not only reduces the expression of drug pumps, but also decreases the secretion of inflammatory factors like CD34 in the tumor microenvironment, thereby increasing the sensitivity of tumor cells to antitumor drugs [27,126].

The impact of miR-665 on drug resistance in cancer treatment highlights the importance of understanding the role of miRNAs in regulating the efficacy of chemotherapy drugs. Further research into the mechanisms by which miR-665 modulates drug resistance may lead to the development of more effective and targeted therapies for cancer.

Though the relationship between miR-665 and radio resistance is still unclear, studies have shown that miR-665 may inhibit cancer radio resistance by targeting key genes involved in the DNA damage response and repair pathway, such as p53 in NSCLC, B-cell lymphoma 2 protein (Bcl-2), which regulates programmed cell death, in hippocampal astrocytes, and ATM in GC [125,147,148], or increase radio resistance by regulating the MAPK pathway, including regulation of NR4A3, SRCIN1, and HOXA10/11 [19,26,149].

## 5. Prospects

MiR-665 is a potentially valuable non-invasive biomarker for cancer development and prognosis. In several cancer types, including breast cancer, cervical cancer, endometrial cancer, colorectal cancer, gastric cancer, and ovarian cancer, higher expression of miR-665 is associated with a decline in patient survival time (based on data from the TCGA database and Kaplan–Meier analysis), and miR-665 in these cases inhibits tumor suppressor genes, like TP53, APC, and BRCA1.

On the other hand, miR-665 is downregulated in retinoblastoma, neuroblastoma, glioma cancer, and cancers of the circulatory system, through multiple molecular mechanisms. This downregulation of miR-665 leads to increased expression of oncogenes like EGFR, BRAF, KRAS, and MYC, which promote tumor cell migration, invasion, and proliferation. In these cancer types, overexpression of miR-665 has been demonstrated to have antiproliferative and proapoptotic effects, suggesting a tumor-suppressive effect. Detecting miR-665 overexpression as soon as possible can in all these cases be a valuable prognostic tool. In the cancer types where it acts as an “oncogene” it can be regarded as a negative marker, whereas in the cancer types where it behaves as a tumor suppressor it is a positive marker.

Understanding the role of miR-665 in cancer can lead to the development of targeted therapies and personalized treatment approaches. The potential impact of microRNAs on patient care is significant. MiR-665’s inclusion in a multianalyte panel of biomarkers can aid in early diagnosis, prognosis prediction, and monitoring of disease progression. Additionally, targeting miR-665 with specific therapies may offer new treatment options for patients with certain cancer types.

In liver cancer and lung cancer, the role of miR-665 is dual and unclear. Survival curves of patients with high expression of miR-665 had distinct, even completely opposite results. In liver cancer, the function of miR-665 differs depending on the subtype of the tumor. In one study [65], including 372 patients with hepatocellular carcinomas, survival time was significantly increased (from 27 months to 81 months) in patients with high miR-665 levels. However, in another study with 167 patients with pan-liver cancer (including HCC, cholangiocarcinoma, gallbladder cancer, intrahepatic bile duct carcinoma, and liver metastasis), survival time was reduced from 76 months to 51 months, suggesting that miR-665 is a lethal factor. So, the precise role of miR-665 in liver cancer is still not fully understood and requires further research.

Concerning lung cancer, research data indicate that miR-665 has a role in promoting cancer. However, Kaplan–Meier data show that patients with high expression of miR-665 seem to have a longer survival period. This is in contrast with clinical data present in the TCGA database that indicate that miR-665 is more expressed in lung cancer tissues compared to normal tissues. This contradiction may be due to a grouping of different subtypes, like LUCD/LUSC, in the database. Therefore, before miR-665 can be used as a diagnostic biomarker for liver or lung cancer more accurate experimental and clinical data are needed [150].

As described above, miR-665 can be considered as a potentially interesting biomarker due to significant expression differences in certain cancer types. However, to be useful in practice miR-665 levels should be easily and quickly measurable. Several methods, like RT-qPCR, have been developed for measuring miR-665 expression. This technique permits the precise and early measurement of miR-665 expression in clinical samples, such as specimen tissues or blood samples [20,128,151], allowing for a more accurate evaluation of tumor risk and prognosis.

Several miR-665-targeted therapeutic approaches are being investigated. These include the use of miR-665 mimics or expression vectors to restore or enhance miR-665 expression and the use of miR-665-targeted siRNAs and shRNAs, among others, to inhibit the expression of cancer-associated genes [19,28,40,88,90].

Nanobodies are small antibody fragments with small size, high stability, and high specificity. They are often used to enhance the therapeutic potential in cancer by conjugating to miRNAs and delivering them to target cells for targeted delivery [152,153]. Also, miRNAs are usually encapsulated in the aqueous core of liposomes or sometimes embedded in bilayer structures. Liposomes protect miRNAs from degradation and facilitate cellular uptake of miRNAs. The specificity of liposomes can be improved by modification of targeting ligands on the liposome surface [154,155].

In addition, miR-665 can potentially be utilized for therapy via inclusion in Extracellular Vesicles (EVs). EVs are lipid bilayer-coated particles, 10 to 500 nm in diameter, that are naturally released from almost all cell types. They are known to facilitate intercellular communication in numerous cellular processes, including immune responses and coagulation. EVs can help disseminate miR-665 throughout the circulatory system, and since EVs are highly biocompatible and low-immunogenic they can serve as a safe and effective treatment. Clinical trials have demonstrated that by loading miR-146b into EVs it can effectively inhibit the proliferation, invasion, and metastasis of glioma cancer cells, and it has been shown to have a good therapeutic effect in rat models [156]. Care should also be taken to avoid side effects caused by the EVs and miR-665. For example, it has been shown that, in both non-small-cell lung cancer (NSCLC) and small-cell lung cancer (SCLC) patients, miR-665 is the most prominent microRNA present in exosomes. This preferential loading of miR-665 seems to be mediated by the higher expression of the lncRNA SCIRT. SCIRT can bind to the heterogeneous nuclear ribonucleoprotein A1 (hnRNPA1), and thus be loaded in EVs together with miR-665 bound to it. MiR-665 released from EVs can target HEYL, a transcription factor acting downstream of Notch, and by doing so promote lung cancer cell invasion and migration [34]. The dual nature of EV-carried miRNAs must be considered when modulating EV levels in patients. Other potential side effects must be considered, as EVs have been shown to be involved in other diseases such as diabetes, fatty liver, and myocardial ischemia [157].

## 6. Conclusions

In conclusion, miR-665 plays diverse roles in different cancer types, and can act either as oncogene or as tumor suppressor. Because of its differential expression between cancer cells in specific types of cancers and normal cells, detecting its levels as soon as possible can be a valuable prognosticss tool for some of them. However, before miR-665 can be applied as a biomarker for certain types of cancers, the conflicting results between some of the clinical and experimental data need to be clarified/eliminated. In addition, the abnormal miR-665 levels reported in certain cancer types need to be confirmed/validated in many more patients (clinical samples) to strengthen the association. The role and molecular interactions of miR-665 in these cancer types need to be elucidated in more detail. Finally, the role of miR-665 in different subtypes of specific cancers needs to be given special attention, because its function may vary depending on the cancer subtype.

## Figures and Tables

**Table 1:** Genes targeted by miR-665.

Target Gene	Cancer Type	Regulatory Function	References
ABCC2	Hepatocellular Cancer; Chronic Myeloid Leukemia	Inhibition of proliferation, invasion, promotion of apoptosis	[67]
AKT/AKT3	Cardiomyocyte Ischemia/Reperfusion Injury; Ovarian Cancer; Gastric Cancer; HEP3B Liver Cancer Cell (HEP3B)	Inhibition of ROS, promotion of viability	[58,68,69]
B7-H3	Breast Cancer	High expression	[70]
Caspase3	Lung Squamous Cell Carcinoma	Inhibition of apoptosis	[20]
CD34	Gastrointestinal Stromal Cancers	Inhibition of migration	[71]
C-MYC	Murine Neuroblastoma	Inhibition of cell cycle	[41]
CRIMI	Gastric Cancer	Inhibition of EMT	[28]
Diaphanous Homolog1	Colorectal Cancer	Inhibition of proliferation	[72]
DVL3	Colorectal Cancer	Inhibition of invasion	[73]
EGFR	Cervical Cancer?	Inhibition of paclitaxel resistance	[74]
ERK and ERK1/2	Bladder Cancer; Acute Lymphoblastic Leukemia; Hepatocellular Carcinoma, Cervical Cancer?	Promotion of proliferation, stemness, inhibition of cancer size	[33,35,60,75]
EXT1/4	Acute Lymphoblastic Leukemia	Promotion of proliferation	[33]
FAK	Gastric Adenocarcinoma Cancer	Promotion of EMT	[45]
GLI2	gastric signet ring cell carcinoma (GSRCC)/IGA	Inhibition of GSRCC/promotion of GADC	[76]
GPX3	Non-Small Cell Lung Cancer	Promotion of glycolysis	[77]
HDAC8	Murine Neuroblastoma	Inhibition of cell cycle	[41]
HEYL	Lung Cancer	Promotion of metastasis	[34]
HIPPO	Hepatocellular Carcinoma	Promotion of migration, invasion	[78]
HMGA1	Glioma	Inhibition of proliferation	[79]
HMGB1/3	Glioma; Neuroblastoma; Tongue Squamous Cell Carcinoma;	Inhibition of migration, invasion, oncogenicity	[38,80,81]
HOXA1	Hepatocellular Cancer	Inhibition of motility	[82]
HOXA10	Ovarian Cancer	Inhibition of proliferation	[83]
HOXB5	Endometrial	Inhibition of colony formation, invasion, migration	[84]
IGF2	Hepatocellular Carcinoma (HCC)	Inhibition of mobility	[85]
LIM	Diffuse Large B Cell Lymphoma (DLBCL)	Inhibition of proliferation	[20]
LLGL1	Small Cell Lung Cancer (SCLC)	Promotion of S-phase fraction	[86]
MAPK/ERK	Hepatocellular Carcinoma (HCC); Gastric Cancer (GC); Lung Cancer	Promotion of proliferation by Evs, inhibition of apoptosis	[27,57,75]
NR4A3	Breast Cancer; Bladder Cancer	Promotion of metastasis, stemness	[19,60]
ORMDL3	Inflammatory bowel disease	Promotion of inflammatory	[87]
PAK1	Cardiomyocyte Ischemia/Reperfusion Injury	Inhibition of ROS, cell apoptosis	[69]
PLCG1	GSRCC/IGA	Inhibition of GSRCC/promotion of GADC	[76]
PPP2R2A	Gastric Cancer (GC)	Inhibition of EMT	[88]
PRICKLE2	Lung Adenocarcinoma	Promotion of migration	[89]
PTPRB	Lung Cancer; Hepatocellular Carcinoma	Promotion of proliferation	[90,91]
RAB23	Osteosarcoma	Inhibition of invasion	[91]
SAMD1/2/3	Cervical; Pancreatic Cancer; Bladder Cancer	Inhibition of cancer size	[32,35,92]
SH3LASP1	Diffuse Large B Cell Lymphoma (DLBCL)	Inhibition of proliferation, invasion. Promotion of apoptosis	[40]
SNAL	Bladder Cancer	Inhibition of EMT	[32]
SOCS3	Gastric Adenocarcinoma Cancer	Promotion of EMT, migration	[45]
SRCIN1	Ovarian Cancer	Promotion of proliferation	[26]
STAT3	Gastric Cancer (GC); Colorectal Cancer; Breast Cancer	Inhibition of proliferation, oncogenicity	[70,93,94]
TGF-bR1/2	Pancreatic Cancer; Cervical Cancer; Chronic Myeloid Leukemia	Inhibition of cell cycle proliferation	[35,92,95]
TREM2	Microglia Cancer	Inhibition of inflammatory processes	[96]
TRIM8	Lung Squamous Cell Carcinoma (LUSC)	Promotion of proliferation	[20]
VEGF-α	Ovarian Cancer	Inhibition of angiogenesis	[97]
Wnt5a/b-catenin	Lung Squamous Cell Carcinoma (LUSC); Retinoblastoma; HEP3B	Inhibition of oncogenicity	[20]
XBP1	Inflammatory bowel disease, Breast Cancer	Promotion of apoptosis	[87]
YAP1	Gastric Cancer (GC)	Inhibition of migration	[98]
YRNA-hY4	NSCLC	High expression	[99]
YWHAZ	GC	Inhibition of viability	[100]

Table 1 shows target genes and functions of miR-665 in different cancers.

## Data Availability

Not applicable.
